# Evolving Evidence-Driven Strategies in Cardiogenic Shock Management

**DOI:** 10.1016/j.jacadv.2026.103225

**Published:** 2026-09-15

**Authors:** Anju Bhardwaj, Dustin Hillerson, Nikhil Narang, Megan N. Pelter, Anjan Sinha, Maya Guglin

**Affiliations:** a University of Virginia, Charlottesville, Virginia, USA; b Mayo Clinic, Rochester, Minnesota, USA; c University of Chicago, Chicago, Illinois, USA; d Loma Linda University School of Medicine, Loma Linda, California, USA; e Indiana University School of Medicine, Indianapolis, Indiana, USA; f Rutgers University, New Brunswick, New Jersey, USA

**Keywords:** cardiogenic shock, clinical guidelines, mechanical circulatory support

## Abstract

Although existing guidelines and expert consensus documents provide a general framework for management of cardiogenic shock (CS), a substantial body of evidence has yet emerged which has not been fully incorporated into current recommendations. This State-of-the Art review synthesizes both established and recent literature to provide an updated perspective on contemporary CS management. We review randomized controlled trials and key observational studies evaluating invasive hemodynamic monitoring, pharmacologic therapies, temporary mechanical circulatory support, and structural and valvular interventions. We critically appraise trial design, patient heterogeneity, and methodological limitations that influence interpretation and clinical applicability. Finally, we highlight persistent gaps and ongoing trials that aim to refine risk stratification and treatment strategies. This review provides a comprehensive, up-to-date synthesis of the CS literature and outlines key directions for future research and clinical practice.

## Highlights

• Emerging evidence has outpaced existing CS guidelines, requiring updated evidence-driven management frameworks.
• This review synthesizes contemporary evidence to guide phenotype-driven CS management.
• Future progress requires refined phenotyping, pragmatic trials, and designs within standardized multidisciplinary shock care pathways.

At least 3 major professional societies have published guidelines that provide evidence-based recommendations on management of cardiogenic shock (CS), with a broadly consistent definition across societies. The American Heart Association (AHA) scientific statement on contemporary management of CS (2017) defined CS as ineffective cardiac output due to a primary cardiac disorder resulting in clinical and biochemical manifestations of inadequate tissue perfusion.^1^ The European Society of Cardiology guidelines (2021) describe CS as a life-threatening syndrome of primary cardiac dysfunction leading to tissue hypoperfusion, multiorgan failure, and death.^2^ Finally, the 2022 AHA/American College of Cardiology (ACC)/Heart Failure Society of America heart failure (HF) guideline further characterizes CS as “a critical reduction in cardiac output manifested by end organ dysfunction”.^3^ Although these guidelines provide an essential framework for CS diagnosis and management, several randomized controlled trials (RCTs) and key studies have emerged since their publication, prompting a reassessment of the contemporary evidence base. More recently, the ACC released a focused clinical guidance document to complement existing recommendations^4^; however, the trial data are largely embedded within a broader clinical narrative, with emphasis on a limited number of select studies. In this review, we synthesize the most recent evidence in CS management and propose evolving best strategies (Central illustration).

**Central Illustration:**
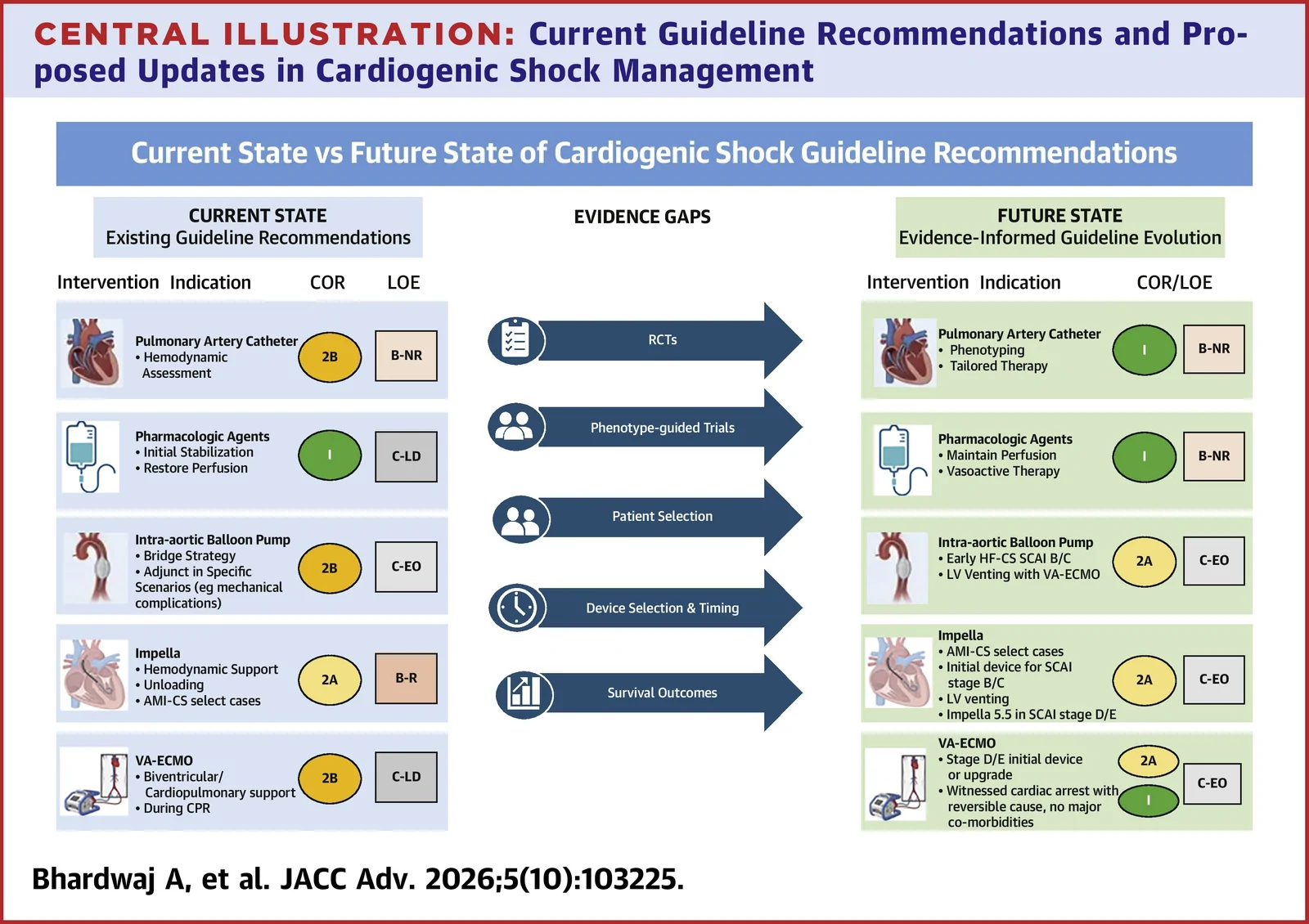
Current Guideline Recommendations and Proposed Updates in Cardiogenic Shock Management Comparison of current guideline recommendations with a proposed future evidence-informed framework for cardiogenic shock management. The center arrows highlight key evidence gaps needed to strengthen future recommendations. AMI-CS = acute myocardial infarction-cardiogenic shock; B-NR = moderate quality evidence from nonrandomized studies; B-R = moderate quality evidence from 1 or more RCT; C-EO = expert opinion; C-LD = limited data; COR = class of recommendation; CPR = cardiopulmonary resuscitation; HF-CS = heart failure–cardiogenic shock; LOE = level of evidence; LV = left ventricle; PAC = pulmonary artery catheter; RCT = randomized controlled trial; SCAI = Society for Cardiovascular Angiography and Interventions; VA-ECMO = venoarterial-extracorporeal membrane oxygenation.

## Invasive hemodynamic monitoring in CS

The use of pulmonary artery catheter (PAC) for invasive hemodynamic monitoring has been extensively studied and debated over the years. Originally invented in the 1920s, it underwent modifications over subsequent decades. By the mid-20th century, PAC became an essential tool in medical practice, particularly before the advent and widespread adoption of echocardiography. Despite this, its use in large patient populations was not documented until 1976.^5^

However, the clinical utility of PAC was called into question in the early 2000s following several RCTs that failed to demonstrate a survival benefit in heterogenous cohorts of critically ill patients. Trials from Canada and France showed no survival advantage with PAC guided care,^6^^,^^7^ and the UK PAC-Man trial similarly reported no differences in in-hospital, intensive care unit (ICU), or 28-day mortality.^8^ Notably, these studies were conducted in heterogenous population of critically ill patients, including those with acute respiratory distress syndrome, mixed medical-surgical ICU cohorts, and high-risk surgical populations, rather than patients with CS. Although CS patients were not the intended focus of these trials and substantial crossover across RCTs confounded results, these studies nevertheless exerted a major influence on clinical practice in HF community.

In 2005, the ESCAPE (Evaluation Study of Congestive Heart Failure and Pulmonary Artery Catheterization Effectiveness) trial^9^ further fueled skepticism by demonstrating no improvement in 6-month survival or hospitalizations with PAC management compared with clinical assessment alone in patients with acutely decompensated HF. Importantly, only 1.8% of patients in ESCAPE met the clinical definition of CS, leaving the role of PACs in true CS largely untested by randomized evidence.

Another key limitation of these early RCTs relates not only to patient selection but also to challenges in interpretation and application of hemodynamic data. These studies were conducted in noncardiac ICU settings, where expertise in waveform analysis and derived indices was variable, and management was often nonprotocolized and left to clinician discretion. These studies have highlighted difficulties in waveform recognition, accurate data interpretation, and inconsistency in translating measurements into therapeutic decisions, all of which may have attenuated potential benefits of PAC use.

In contrast, contemporary CS management is increasingly delivered in specialized cardiac ICUs and multidisciplinary shock teams, where proficiency in invasive hemodynamic interpretation is greater and integrated into structured clinical decision making. PAC data are not used in isolation but combined with imaging and clinical assessment to guide phenotype-specific therapy and escalation to temporary mechanical circulatory support (tMCS), potentially mitigating many of the limitations noted in earlier trials.

### Current guidelines

Reflecting this uncertainty, contemporary guidelines classify PAC use in CS as a Class IIb recommendation, indicating it *may be* considered for select patients (Table 1).

**Table 1:** Current Recommendation on the Use of Pulmonary Artery Catheter in Cardiogenic Shock

Document	Recommendation	Strength	Level of Evidence
AHA, 2017^1^	We suggest the use of PACs in cases of diagnostic or CS management uncertainty or in patients with moderate to severe CS who are unresponsive to initial therapy. Hemodynamic monitoring should complement (and not replace) other markers of end-organ perfusion in CS.		
ESC guidelines, 2021^2^	No recommendations in the main body of the Guidelines. Per supplemental material, PAC may be considered in patients who, despite pharmacological interventions, remain refractory as the severity of illness can be underestimated when relying on clinical features alone.		
AHA/ACC/HFSA, 2022^3^	In patients presenting with CS, placement of a PAC may be considered to define hemodynamic subsets and appropriate management strategies	2B	B-NR

ACC = American College of Cardiology; AHA = American Heart Association; B-NR = moderate quality evidence from nonrandomized studies; CS = cardiogenic shock; ESC = European society of Cardiology; HFSA = Heart Failure Society of America; PAC = pulmonary artery catheter.

### Contemporary clinical evidence

Since ESCAPE, numerous observational, retrospective, pragmatic studies and meta-analyses have readdressed the use of PAC in CS across all Society for Cardiovascular Angiography and Interventions (SCAI) stages, including patients on tMCS. Hernandez et al^10^ reported a temporal decline in PAC use from 2004 to 2014; however, PAC use in CS patients was associated with lower in-hospital mortality (35.1% vs 39.2%; *P* < 0.001) and fewer cardiac arrests (14.9% vs 18.3%; *P* < 0.001), whereas paradoxically higher mortality (9.9% vs 3.3%) was observed among non-CS HF patients receiving PACs. This observational study is one of several retrospective and observational analyses demonstrating mortality benefit in CS patients. This divergence underscores the context-specific utility of invasive hemodynamic monitoring, suggesting that its benefit is more pronounced is CS, rather than in broader HF populations.

The apparent contradiction to prior RCTs may reflect either improved patient selection or advances in clinical management, but it should be interpreted with caution, given the inherent limitations of observational data. Importantly, these contemporary findings likely reflect a shift from indiscriminate PAC use toward targeted deployment within structured, shock-team driven care models, rather than a simple intervention effect.

Smaller, observational studies have paved the way to larger registry databases evaluating the use of PAC in CS. A pragmatic observational study using real-world data from the Cardiogenic Shock Working Group (CSWG)^11^ demonstrated higher in-hospital mortality in patients who did not undergo PAC assessment compared to those who had complete hemodynamic profiling (*P* < 0.001), with consistent associations observed across SCAI stages. These findings were corroborated by Critical Care Cardiology Network registry,^12^ PAC use was associated with improved survival in CS (OR: 0.79; 95% CI: 0.66-0.96; *P* = 0.017). Notably, substantial interinstitutional variability in PAC utilization was observed, underscoring the influence of institutional protocols and shock team expertise.

A single-center Canadian registry^13^ of 1043 CS patients further demonstrated that PAC use was associated with a greater use of tMCS) and lower in-hospital mortality (29.3% vs 36.2%; *P* = 0.02) with the strongest benefit observed in SCAI stages D-E. Another study from CSWG^14^ focused on HF-related CS (HF-CS) patients showed that PAC use was associated with lower in-hospital mortality (22.2% vs 29.8%; OR: 0.68; 95% CI: 0.50-0.94), particularly when implemented early (within 6 hours of admission), compared with delayed (≥48 hours) or no PAC use (17.3% vs 27.7%; OR: 0.54; 95% CI: 0.37-0.81).

Collectively, these observational data challenge the continued class IIb status of PAC in current guidelines, which are largely extrapolated from non-CS patients and historical ICU practices. In contemporary CS care, PAC-guided profiling is increasingly central to phenotype driven management, particularly in advanced CS patients where clinical assessment alone is insufficient to guide therapy, and when instituted early. However, such findings must be interpreted with caution, as confounding by indication is substantial, as patients selected for PAC monitoring often reflect clinician’s prognostic expectations, and intensity of care (early escalation to tMCS) and institutional practice patterns (presence of multidisciplinary shock teams), which independently shape outcomes. Therefore, PAC use may function as a marker of high-quality, protocolized care rather than an isolated therapeutic intervention.

From a mechanistic standpoint, PAC enables early, continuous, and quantitative hemodynamic phenotyping beyond what bedside assessment or echocardiography can reliably provide. Direct measurement of variables including cardiac output and filling pressures, with a PAC, facilitates precise phenotyping and targeted therapeutic intervention, timely escalation of support, and allows dynamic reassessment.

When implemented early within experienced, protocol-driven multidisciplinary shock teams, this physiology-based approach may reduce exposure to ineffective or harmful therapies, mitigate irreversible end-organ injury, and ultimately translate into improved outcomes. Nevertheless, these mechanistic explanations remain inferential, and untested in randomized frameworks.

#### Future directions

Rather than evaluating PAC as a binary intervention, future trials should focus on hemodynamic-guided treatment strategies, where invasive monitoring is embedded within predefined escalation algorithms. Adaptive or platform trial designs that stratify patients by phenotype and timing of intervention will be critical to determine whether PAC-guided care improves outcomes.

## Pharmacologic management in CS

Initial management of CS relies on the use of vasoactive and inotropic medications to restore perfusion and prevent or reverse or end-organ dysfunction. However, robust RCTs directly addressing pharmacological management are limited and the use of vasoactive medications in CS is predominantly guided by small RCTs, observational studies, consensus statements by clinical experts or extrapolated from studies from acutely decompensated HF and other shock states, each with important limitations (Table 2). For instance, the OPTIME-CHF (Outcomes of a Prospective Trial of Intravenous Milrinone for Exacerbations of Chronic Heart Failure) trial^15^ evaluated intravenous milrinone in acutely decompensated HF but excluded patients with CS, highlighting the persistent reliance on indirect evidence to inform management of a distinct and hemodynamically complex syndrome.

**Table 2:** Current Recommendation on the Use of Pharmacological Agents in Cardiogenic Shock

Document	Recommendation	Strength	Level of Evidence
AHA, 2017^1^	Consider using early in the treatment course in patients not responsive to initial therapy or in cases of diagnostic or therapeutic uncertainty		
ESC guidelines, 2021^2^	Inotropic agents may be considered in patients with SBP <90 mmHg and evidence of hypoperfusion who do not respond to standard treatment, including fluid challenge, to improve peripheral perfusion and maintain end-organ function	IIB	C
	A vasopressor, preferably norepinephrine, may be considered in patients with cardiogenic shock to increase blood pressure and vital organ perfusion.	IIB	B
	Inotropic agents are not recommended routinely, due to safety concerns, unless the patient has symptomatic hypotension and evidence of hypoperfusion.	III	C
AHA/ACC/HFSA, 2022^3^	In patients with cardiogenic shock, intravenous inotropic support should be used to maintain systemic perfusion and preserve end-organ performance.	1	B-NR

SBP = systolic blood pressure; other abbreviations as in Table 1.

The earliest RCT to evaluate superiority of one vasopressor agent over another in shock was the SOAP II (Sepsis Occurrence in the Acutely ill Patients) trial.^16^ This multicenter, large trial comparing dopamine to norepinephrine in critically ill patients, demonstrated higher arrhythmia burden and increased 28-day mortality with dopamine, highlighting norepinephrine’s superior safety profile. Although CS patients were not stratified, the data remains clinically informative positioning norepinephrine as the preferred first-line therapy, with de-emphasis on dopamine use in CS management. Small limited RCTs evaluating epinephrine in CS demonstrated increased lactate levels, metabolic derangements, and arrhythmic complications without clinical benefit, further emphasizing norepinephrine as the preferred agent.^17^^,^^18^ Levosimendan has shown promise in select acute myocardial infarction–related CS (AMI-CS) studies, but results remain heterogenous and interpretation is limited by small sample sizes, variable study design, and lack of availability in certain regions, including North America.^19^^,^^20^

### Current guidelines

Current international guidelines reflect the limitations of the evidence base and lack of mortality benefit with use of inotropes and recommend norepinephrine as first-line vasopressor therapy in CS, with inotropes reserved for low-output states and selected phenotypes (Table 2). Notably, these recommendations do not incorporate systematic hemodynamic phenotyping or protocolized treatment pathways, which are increasingly central to contemporary CS care in specialized centers.

### Contemporary clinical evidence

Randomized CS specific evidence of inotropes remains sparse. The DOREMI (Dobutamine Compared with Milrinone) trial^21^ randomized 192 CS patients to receive milrinone or dobutamine, and found no difference in composite endpoint encompassing all-cause mortality, major adverse cardiovascular events, and organ failure suggesting no clear superiority between the 2 agents. The trial was methodologically rigorous, but interpretation is constrained by modest sample size, clinical heterogeneity, nonstandardized dosages, limited follow-up, and use of broad composite outcomes.

These neutral results cannot be equated with therapeutic equivalence. Rather, this trial highlights the intrinsic limitations of evaluating 2 pharmacologic agents within a highly heterogenous syndrome. The enrollment in this trial was broadly based on clinical criteria across a broad spectrum of SCAI stages, without systematic invasive hemodynamic characterization or phenotyping stratification, thereby aggregating patients with fundamentally distinct pathophysiology and therapeutic needs. As a result, potential differential treatment effects within specific phenotypes may have been obscured.

In addition, inotrope selection, dosing, and escalation were guided by clinician discretion rather than a protocolized or physiology-based paradigm, effectively testing variable, nonstandardized therapeutic approaches rather than a defined treatment strategy. The inclusion of composite endpoints incorporating downstream outcomes such as tMCS use further introduces variability driven by institutional practice patterns rather than pharmacologic efficacy.

Therefore, results of DOREMI did not meaningfully alter clinical practice, as inotrope selection remains contextual, phenotype-dependent, and guided by hemodynamic goals. Albeit, the trial highlights a broader conceptual limitation in CS research; and comparison of inotropes are unlikely to yield actionable insights in the absence of physiologic stratification and structured treatment algorithms.

The study also underscores the absence of RCTs evaluating early, phenotype-specific, and protocolized inotrope strategies integrated with invasive hemodynamic assessment and predefined escalation pathways. In a post hoc analysis, milrinone was associated with improved outcomes among patients without acute kidney injury, whereas no benefit was observed in those who developed renal failure, further emphasizing the importance of patient selection and end-organ function in therapeutic response.^22^

Overall, contemporary pharmacologic management of CS continues to rely on low-moderate quality evidence, with no agent demonstrating clear superiority.

#### Future directions

Advancing pharmacologic management in CS will require a shift from agent level comparisons and evaluate hemodynamic-guided, phenotype-specific treatment strategies, integrating early invasive assessment with protocolized escalation pathways.

## Intra-aortic balloon pump in CS

First introduced in 1968, intra-aortic balloon pump (IABP) remains the oldest and most commonly used tMCS device.^23^^,^^24^ Early physiologic studies demonstrated improvement in cardiac output and coronary perfusion,^25^ supporting its initial adoption in AMI-CS.^26^ However, RCTs both pre-reperfusion and postreperfusion consistently failed to demonstrate improvement in survival or sustained hemodynamics^27^, ^28^, ^29^, ^30^ Among these, in the small IABP SHOCK trial, adding IABP did not significantly improve multiorgan dysfunction, hemodynamics, or markers of illness severity compared with standard care alone.^30^ The follow-up landmark IABP-SHOCK II trial in 2012 randomized 598 AMI-CS patients to IABP during percutaneous coronary intervention (PCI) or medical therapy alone. There was no significant mortality difference between the 2 groups with 39.7% mortality in the IABP arm vs 41.3% mortality in the control arm (relative risk: 0.96; 95% CI: 0.79-1.17; *P* = 0.69).^29^ Interestingly, this trial evaluated a device with modest hemodynamic support in a population where myocardial salvage with revascularization is the primary determinant of outcomes, without integration into a protocolized or hemodynamically guided treatment strategy. Other RCTs comparing the IABP with alternative tMCS devices in AMI-CS did not show any survival benefit, although these studies were small and not powered to evaluate hard endpoints.^31^^,^^32^

Following the IABP-SHOCK II trial, IABP use in AMI-CS declined by approximately 50%,^33^ yet contemporary registry data demonstrated continued real-world use, reflecting its safety profile, deployment ease, and limited alternatives in select scenarios.^34^ Importantly, across all AMI-CS trials, IABP failed to meaningfully improve invasive hemodynamics, which may in part reflect late deployment, high vasopressor burden, and advanced shock severity at the time of initiation.

In contrast, HF-CS is characterized by chronically elevated afterload and impaired ventricular-arterial coupling, providing a stronger mechanistic rationale for IABP support.^35^ Accordingly, IABP remains the most frequently used tMCS device in HF-CS, utilized in approximately 13% of hospitalizations, with reported mortality rates ranging from 13% to 41%.^36^, ^37^, ^38^, ^39^, ^40^ It is commonly employed as a bridge to durable left ventricular assist device (LVAD) or heart transplantation (HT).^41^^,^^42^ The ALT-SHOCK-1 phase II trial^43^ prospectively assessed IABP support in combination with inotropes in 24 HF-CS patients and reported a 60-day mortality of 12.5%. In the only RCT comparing IABP to inotropes alone, IABP use was associated with early and greater improvements in mixed venous oxygen saturation, cardiac power output, natriuretic peptide reduction, fluid balance, and dyspnea severity at 3 hours.^36^ Although 30-day mortality was numerically lower with IABP (23% vs 44%), the trial remains hypothesis-generating given the single arm design, limited sample size, and lack of statistical power.

Response to IABP in HF-CS appears highly dependent on patient selection. Favorable predictors include nonischemic cardiomyopathy, higher left ventricular cardiac power index, elevated systemic vascular resistance, significant mitral regurgitation (MR), elevated filling pressures, preserved right ventricular function, and early implantation.^37^^,^^38^^,^^41^^,^^44^^,^^45^ Conversely, advanced right ventricular dysfunction and elevated peripheral vascular resistance may attenuate hemodynamic benefit.^41^^,^^46^ These observations collectively suggest that clinical effectiveness of IABP is not uniform, but contingent on underlying hemodynamic phenotype and timing of deployment.

### Current guidelines

Current guidelines assign IABP a Class IIb recommendation in CS, reflecting equipoise and absence of definitive randomized evidence demonstrating survival benefit. Guideline recommendations are summarized in Table 3. However, this classification is largely derived from AMI-CS dominant trial populations and may not capture its role in selected HF-CS phenotypes or within contemporary, protocol-driven CS care models.

**Table 3:** Current Recommendations on the Use of Intra-aortic Balloon Pump in Cardiogenic Shock

Document	Recommendation	Strength	Level of Evidence
AHA, 2017^2^	IABP can be considered in patients with CS with acute mitral regurgitation or a ventricular septal defect, and it can be considered in select patients with profound CS when other MCS devices are not available, are contraindicated, or cannot be placed.		
ESC, 2021^3^	IABP may be considered in patients with CS as a bridge to recovery, bridge to decision, bridge to bridge, including treatment of the cause of CS (i.e. mechanical complication of acute MI) or long term MCS or transplantation.	IIB	C
	IABP is not routinely recommended in post-MI CS	III	B
AHA/ACC/HFSA, 2022^4^	In patients with CS, temporary MCS is reasonable when end-organ function cannot be maintained by pharmacologic means to support cardiac function	2a	B-NR

IABP = intra-aortic balloon pump; MCS = mechanical circulatory support; MI = myocardial infarction; other abbreviations as in Table 1.

### Contemporary clinical evidence

Since the release of clinical guidelines, several retrospective studies have compared IABP with other tMCS devices across different CS etiologies, with most results favoring IABP^47^, ^48^, ^49^, ^50^, ^51^ (Table 4). However, these findings are subject to substantial confounding by indication, institutional practice patterns, and differences in escalation strategies. In a study by Jentzer et al^52^ involving 934 CS patients (60% AMI-CS), in-hospital mortality was significantly lower in the 39% who received IABP (27% vs 43%). Mortality reduction was observed across all SCAI shock stages (*P* < 0.05, except stage E). Similar findings linking IABP to lower mortality in CS were reported by Luo et al^53^ and Yuan et al.^54^

**Table 4:** Clinical Trials of Temporary Mechanically Circulatory Support Devices

	Type of Shock	MCS Devices	Primary/Key Outcomes	Key Limitations
IABP-SHOCK II^29^	AMI-CS	IABP vs SOC	No reduction in 30-day, 1-year or long-term mortality	Late device placement, minimal hemodynamic profiling; exclusion of mechanical complications, reflects preshock team era
IABP-SHOCK I^28^	AMI-CS	IABP vs SOC	No mortality benefit	Small sample size; early-generation care; limited power
IMPRESS^56^	Severe AMI-CS	Impella CP vs IABP	No difference in 30-day or 6-month mortality; very high mortality in both arms	Small trial, profound shock, limited external validity
IMPELLA-STIC^57^	AMI-CS	Impella LP5.0 + IABP vs IABP alone	No improvement in mortality or hemodynamics	Early termination; underpowered; specific subset of AMI-Cs patients
DanGer Shock^60^	AMI-CS	Impella CP vs SOC	13% absolute reduction in 180 day all-cause mortality in Impella arm compared to SOC (45.8% vs 58.5%; *P* = 0.04)	Highly selective enrollment; treatment at experienced center; Nearly half received Impella device before PCI, limited generalizability
EUROSHOCK^67^	AMI-CS	VA-ECMO vs SOC	30-day mortality 43.8% in VA-ECMO arm vs 61.1% in SOC arm (HR: 0.56; *P* = 0.22) 1-year all-cause mortality 51.8% in VA-ECMO group to 81.5% in SOC arm (HR: 0.52; *P* = 0.14)	Pilot trial, underpowered, heterogeneity in shock severity and timing
ECMO-CS^68^	AMI-CS	VA-ECMO vs SOC	No significant reduction in 30-day mortality	Crossover allowed, delayed ECMO initiation, increased bleeding and vascular complications, limited generalizability, small sample size
ECLS-SHOCK^69^	Acute MI	Early VA-ECMO vs SOC	No reduction in 30-day mortality, higher complication rate	Broad inclusion criteria; heterogeneity, deployment; lack of stratification of shock phenotypes
ALT-SHOCK-2^43^	HF-CS	IABP vs SOC	No definitive mortality benefit	Underpowered, heterogenous HF-CS; evolving background therapy
ISAR-SHOCK^32^	Acute MI	Impella LP 2.5 vs IABP	Impella is safe, superior hemodynamic support but similar mortality	Small size, smaller, early generation pump

AMI-CS = acute myocardial infarction–cardiogenic shock; HF-CS = heart failure–cardiogenic shock; PCI = percutaneous coronary intervention; SOC = standard of care; VA-ECMO = venoarterial-extracorporeal membrane oxygenation; other abbreviations as in Table 3.

Most recently, the multicenter Altshock-2 trial^55^ marked a significant and unanticipated development, where IABP failed to improve 60-day survival or bridging to HT and LVAD in patients with HF-CS (SCAI stages B-D) compared to standard care. The study randomized 101 patients with HF-CS to IABP plus standard care or standard care across 7 centers in Italy over 4 years. Shock severity by SCAI staging was B in 28%, C in 57%, and D in 15%. The trial was halted early for futility at interim analysis. However, interpretation of these findings requires careful consideration of key limitations, including high exclusion rates (>90%),small sample size combined with 13% crossover rate, and inclusion of a high proportion of lower-risk (SCAI-B) patients, all of which may have attenuated the ability to detect benefit. In addition, low baseline lactate levels, and high survival in the control arm raise concerns about suboptimal patient selection. Additional concerns include the lack of standardization in use of IABP and inotropes, with minimal use of PACs, which may not reflect current CS management practices in specialized shock centers.

Taken together, these data suggest that the role of IABP in CS is not uniformly limited, but highly dependent on patient selection, timing of deployment, and underlying hemodynamic phenotype. In particular, early use in selected HF-CS phenotypes with preserved right ventricular function and elevated afterload may represent a more rational application rather than indiscriminate use in AMI-CS. These findings also underscore the limitations of current trial designs and highlight the need for robust, pragmatic, multicenter RCTs evaluating phenotype-driven tMCS strategies guided by invasive hemodynamics and structured protocols.

#### Future directions

Future trials should avoid device vs device comparisons and rather focus on strategy-based approaches, where IABP may be deployed in predefined phenotypes and integrated within structured escalation pathways.

## Impella in CS

The Impella (Johnson & Johnson) device is a microaxial, continuous flow, transvalvular pump that directly unloads the left ventricle. The most used configurations available are Impella CP (3.5 L/min flow) and Impella 5.5 (5-6 L/min flow). Outcome and safety data have emerged regarding the efficacy of the Impella CP device in AMI-CS. Despite strong physiologic rationale, clinical outcome data particularly in AMI-CS has been mixed.

In 2017, the IMPRESS (Impella in Severe Shock) trial aimed to determine whether Impella CP reduced 30-day mortality compared to IABP in patients with AMI-CS undergoing primary PCI.^56^ This small, open-label, multicenter RCT of 48 patients with AMI-CS found no difference in 30-day mortality in patients treated by IABP or Impella CP device. The study enrolled an extremely high risk, advanced-stage population characterized by near-universal cardiac arrest, prolonged resuscitation and a substantial burden of anoxic injury; in such settings initiation of hemodynamic support after irreversible injury is unlikely to meaningfully alter outcomes. Moreover, device initiation was nonprotocolized, with no standardized timing or hemodynamic guidance, effectively testing delayed, nonphenotype-guided support rather than an early, targeted intervention. The current trial paradigms fail to capture the time sensitive and physiology dependent nature of effective CS management, highlighting the need of protocol driven strategy, including pre-revascularization support, invasive hemodynamic profiling, and predefined escalation pathways. In a large retrospective analysis of the Impella-CP in AMI-CS by Dhruva et al^34^ using propensity matching, there was an observed higher risk of in-hospital mortality with Impella (45.0%) compared to IABP (34.1%) (absolute risk difference 10.9%; 95% CI: 7.6-14.2; *P* < 0.001). The Impella group also had a higher risk of major in-hospital bleeding (31.3% vs 16.0%; absolute risk difference 15.4%; 95% CI: 12.5-18.2; *P* < 0.001). Further details of observational and smaller prospective analysis comparing Impella vs IABP are summarized in Table 4). A meta-analysis of randomized trials^58^ comparing either TandemHeart or Impella tMCS to IABP showed improved hemodynamics and perfusion markers, without any mortality benefit. However, these trials consistently reported higher vascular access site bleeding with tMCS devices compared to IABP. Another small RCT comparing an older generation Impella 2.5 with IABP failed to show any survival benefit.^32^

### Current guidelines

Until recently, guideline recommendations for Impella use in CS were limited and nonspecific. The 2025 acute coronary syndrome guidelines introduced a class IIa recommendation for Impella CP in selected patients with AMI-CS in the setting of ST-segment elevation AMI^59^ (Table 5).

**Table 5:** Current Recommendations on the Use of Impella (Microaxial Flow Pumps) in Cardiogenic Shock

Document	Recommendation	Strength	Level of Evidence
AHA, 2017^2^	Percutaneous VADs (including microaxial flow pumps) may be considered in patients with refractory cardiogenic shock (CS) despite pharmacologic therapy, when rapid hemodynamic support is required.	IIB	B-NR
ESC, 2021^3^	Short-term mechanical circulatory support devices, including microaxial flow pumps, may be considered in selected patients with CS as a bridge to recovery, decision, or further therapy.	IIB	C
	Routine use of percutaneous VADs in postmyocardial infarction CS is not recommended outside of select cases.	III	B
AHA/ACC/HFSA, 2022^4^	In patients with CS, temporary MCS, including microaxial flow pumps, is reasonable when end-organ function cannot be maintained by pharmacologic means alone.	2a	B-NR
AHA/ACC//ACEP/NAEMSP/SCAI, 2025^38^	In selected patients with STEMI and severe or refractory CS, insertion of a microaxial intravascular flow pump is reasonable to reduce mortality when done at experienced centers	2A	B-R

ACEP = American College of Emergency Phycisians; B-R = moderate quality evidence from 1 or more RCT; NAEMSP = National Association of Emergency Medical Service Physicians; SCAI = Society for Cardiovascular Angiography and Interventions; STEMI = ST-segment elevation myocardial infarction; VAD = ventricular assist device; other abbreviations as in Tables 1 and 3.

### Contemporary clinical evidence

The Danish-German Cardiogenic Shock (DanGer Shock) trial, a contemporary RCT by Moller et al,^60^ evaluated the use of Impella CP in patients with AMI-CS. This multicenter study enrolled 355 patients over 10 years, showing a significant reduction in 180 day-mortality compared with standard care (48.5% vs 58.5%; HR: 0.74; 95% CI: 0.55-0.99; *P* = 0.04). Notably, patients with out-of-hospital cardiac arrest and severe neurologic impairment were excluded, contributing to a high rate of screen failures and limited generalizability to broader AMI-CS populations. In addition, no significant difference was observed in 30-day mortality, which is a common endpoint for CS trials. Importantly, the efficacy of the intervention must be understood in the context of a substantial burden of adverse events in the intervention arm including considerably higher rates of acute kidney injury requiring renal replacement therapy, major bleeding, sepsis, and limb ischemia. Furthermore, rates of bridging to advanced therapies were low, reflecting a relatively selected study population. Although this trial represents the first notion of evidence supporting a specific type of tMCS in AMI-CS management, this must be interpreted cautiously given safety considerations, trial design, and limited external validity.^61^^,^^62^ Standardization of care protocols for Impella CP, particularly emulating practices from high-performing centers involved in the trial, may help optimize outcomes in broader clinical settings.

Following the release of DanGer Shock results, enrollment in the RECOVER IV (Early Impella Support in Patients With ST-Segment Elevation Myocardial Infarction Complicated by Cardiogenic Shock) trial was halted. Notably, a Critical Care Cardiology Network registry analysis suggested that only a minority of real-world AMI-CS patients would meet DanGer Shock inclusion criteria, further constraining external validity.^63^ The recent 2025 ACC/AHA/American College of Emergency Physicians/National Association of Emergency Medical Service Physicians-SCAI guidelines for acute coronary syndromes have recognized Impella CP as a tMCS option for AMI-CS patients in refractory cases, granting a 2A class of recommendation.^59^

Although Impella in AMI-CS has been studied, data on its use in HF-CS remain limited. The Impella 5.5 device is emerging as a bridge to HT and durable LVAD. The largest contemporary data set on Impella 5.0 and 5.5 in 484 HF-CS patients reported that 25.2% (N = 122) were bridged to LVAD and 32.6% (N = 157) to HT.^64^ Given placement in the axillary artery via surgical cutdown, incidence of limb ischemia (5%, N = 24) was lower compared to Impella CP devices but major bleeding occured in 39% (N = 189) of patients, particularly in those who had additional tMCS devices such as venoarterial extracorporeal membrane oxygenation (VA-ECMO). Additional large-scale analysis from the CSWG studying the time duration of 14 days and beyond for patients supported by the Impella 5.5 concluded no significantly higher reported risk of serious adverse events in patients supported by the device beyond 14 days compared to patients with shorter device duration.^65^

In summary, Impella provides effective unloading and short-term hemodynamic support, but survival benefit remains inconsistent and highly dependent on patient selection, timing, and center experience. Its use in CS should be individualized, balancing physiologic benefit against a substantial burden of device-related complications, and ideally guided by multidisciplinary shock teams and invasive hemodynamic monitoring.

#### Future directions

The key unanswered question is not whether Impella works, but in whom, when, and as part of which strategy. Future trials should focus on early, pre-revascularization deployment, hemodynamic-guided escalation, and integration within multidisciplinary shock care pathways.

## VA-ECMO in CS

VA-ECMO provides full cardiopulmonary support and has been investigated in CS, but early randomized evidence was limited and underpowered. The first RCT^66^ evaluating extracorporeal life support (ECLS) in AMI-CS randomized 42 patients to ECLS or standard of care and demonstrated feasibility but no significant improvement in left ventricular ejection fraction and mortality at 30 days. Preserved left ventricular ejection fraction in both arms may have reflected postcardiac arrest syndrome, and baseline imbalances, limiting any definitive conclusions. Importantly, this trial should not be interpreted as evidence against ECLS in CS, but rather it highlighted the critical challenges in trial design in this domain like patient selection, timing of intervention, endpoint selection, and integration of adjunctive therapies, and underscored the need for adequately powered phenotype-specific RCTs focusing on survival.

The EURO-SHOCK (Testing the Value of Novel Strategy and Its Cost Efficacy in Order to Improve the Poor Outcomes in Cardiogenic Shock)^67^ trial, designed to assess early VA-ECMO in AMI-CS after primary PCI. The trial was terminated prematurely after enrolling only 33 AMI-CS patients, only 13% of screened patients, underscoring the difficulty in identifying an appropriate and generalizable population in CS. Although 30-day mortality appeared lower with VA-ECMO (44% and 61%), this difference was not statistically significant.^67^ Furthermore, although termed early support, the median time to VA-ECMO initiation approached 5 hours from shock onset, suggesting that future investigations must focus on earlier initiation strategies after weighing against the risk of vascular complications.

### Current guidelines

Current guidelines (Table 6) offer cautious recommendations for VA-ECMO in CS, reflecting limited high-quality evidence and high risk of complications The use is generally reserved for refractory CS with rapidly deteriorating hemodynamics or as a bridge to recovery or advanced therapies.

**Table 6:** Current Recommendations on the Use of Venoarterial Extracorporeal Membrane Oxygenation in Cardiogenic Shock

Document	Recommendation	Strength	Level of Evidence
AHA, 2017^2^	Venoarterial ECMO may be the preferred temporary MCS option when there is poor oxygenation that is not expected to rapidly improve with an alternative temporary MCS device or during cardiopulmonary resuscitation		
	Short-term MCS should be considered in patients with cardiogenic shock as a bridge to recovery, bridge to decision, bridge to bridge.	IIB	C
AHA/ACC/HFSA, 2022^4^	In patients with cardiogenic shock, temporary MCS is reasonable when end-organ function cannot be maintained by pharmacologic means to support cardiac function	2a	B-NR

ECMO = extracorporeal membrane oxygenation; other abbreviations as in Tables 1 and 3.

### Contemporary clinical evidence

Several RCTs on VA-ECMO in CS have been published recently. The multicenter ECMO-CS^68^ trial randomized 117 patients with advanced CS (SCAI stage D-E) of mixed etiologies to immediate VA-ECMO or standard care. No differences were observed in the composite endpoint of death, another MCS, or resuscitated cardiac arrest at 30 days (63.8% vs 71.2%), with similar -mortality (50% vs 47.5%) and serious adverse events (60.3% vs 61.0%). However, its neutral outcomes require careful contextualization. A high crossover rate (39%) to VA-ECMO from conservative arm, heterogenous etiologies, lack of protocolized startegies, and frequent complications (>60% in each arm) likely influenced outcomes. Although the investigators concluded that early VA-ECMO in patients with rapidly deteriorating or severe CS did not improve clinical outcomes,^68^ these findings reflect limitations in trial design, patient selection, and integration of adjunctive therapies.

Another RCT, the ECLS-SHOCK trial^69^ focused on AMI-CS patients, deemed less critically ill (50% SCAI stage C). Most (77.7%) patients underwent PCI. At 30 days, mortality was similar between VA-ECMO and control groups (47.8% vs 49%). Bleeding (23.4% vs 9.6%) and vascular complications (11.0% vs 3.8%) were higher with VA-ECMO, potentially offsetting any physiological benefit. Furthermore, left ventricular venting was infrequently utilized (5.8%), and the inclusion of high proportion of resuscitated patients (77.7%) with competing risks such as anoxic brain injury may have further attenuated the likelihood of demonstrating survival benefit. Also, as known from SHOCK trial,^70^ early revascularization improves survival in AMI-CS; therefore, simultaneous PCI or immediately after ECMO may explain short time on ECMO support.

Adjunctive left ventricular unloading strategies during VA-ECMO have been explored. The EARLY-UNLOAD trial,^71^ a prospective, multicenter RCT evaluated Impella for active left ventricular unloading in VA-ECMO patients, found no 30-day mortality benefit, which should be interpreted in the context of key limitations, including small sample size, single-center design, and allowance of crossover to rescue unloading, which likely attenuated the differences. The use of trans-septal left atrial cannulation as unloading strategy may limit generalizability to use of other modalities like Impella or IABP. These findings do not refute the pathophysiologic rationale for left ventricular unloading, rather suggest that timing, modality, and patient selection may be the critical determinants of benefit.

Finally, The EVOLVE-ECMO trial,^72^ randomized CS patients on femoral VA-ECMO with established left ventricular distension to simultaneous left ventricular venting vs no immediate venting. Although pulmonary congestion improved in the immediate arm, the weaning rate, which was the primary outcome, was similar underscoring that adjunctive strategies may enhance physiology without definitively altering outcomes. These findings should be interpreted in light of key limitations like small sample size, pilot design, and substantial heterogeneity. The choice of ECMO weaning as a primary endpoint may also incompletely capture true myocardial recovery or survival benefit. Larger, adequately powered RCTs are required to determine whether specific phenotypes derive benfit from early, standardized unloading strategies.

Overall, contemporary RCT evidence suggests that although VA-ECMO can stabilize hemodynamics in CS, it has not yet translated into consistent survival benefit. Outcomes are strongly influenced by patient selection, timing, severity of CS, left ventricular unloading strategies, and ECMO-related complications, emphasizing the need for precision use within experienced multidisciplinary teams and further adequately powered trials.

The LEVOECMO trial^73^ evaluated early use of levosimendan in patients with VA-ECMO–supported CS and found no improvement in ECMO weaning at 30 days compared to placebo (68.3% vs 68.3%), with no difference in mortality or other outcomes. These neutral findings are in stark contrast with observational data and suggest that pharmacologic inotropic support may have limited impact in the VA-ECMO setting. Future directions: Future ECMO based trials must adopt early, phenotype specific enrollment, incorporate standardized left ventricular unloading strategies, and evaluate ECMO within protocolized care pathways rather than isolated interventions.

## Contemporary evidence of valvular interventions in CS

MR in the setting of CS is rarely the sole driver of hemodynamic collapse but can substantially worsen hemodynamics by further reducing effective forward flow. Acute severe MR may result from papillary muscle or chordal rupture after AMI, endocarditis or degenerative disease, and may slow reflect functional MR in severe left ventricular dysfunction. In these settings, MR acts as a hemodynamic amplifier rather than a primary CS driver. With increasing availability, transcatheter edge-to-edge repair has emerged as a feasible rescue strategy in selected CS patients with severe MR. Although limited to multicenter registry data, some studies demonstrate improved outcomes when procedural success is achieved.^74^^,^^75^

Evidence supporting transcatheter aortic valve replacement in severe aortic stenosis complicated with CS is similarly observational. Although no randomized data exist, registry data suggest potential survival benefit in selected patients compared with medical therapy alone,^76^^,^^77^ although interpretation is limited by heterogeneity in shock severity, timing of intervention, and adjunctive tMCS.

Overall, transcatheter valvular interventions represent a promising but incompletely defined therapeutic strategy in CS. Current evidence supports feasibility and potential benefit in carefully selected patients, but definitive conclusions await prospective trials integrating shock severity, hemodynamic phenotyping, and standardized support pathways. Some European societies have already advocated transcatheter aortic valve replacement in select groups of aortic stenosis patients with CS,^78^ although prospective randomized data are sorely lacking.

## Future: clinical trials in progress

Numerous clinical trials are currently underway to investigate the management of CS, which are discussed in Table 7.

**Table 7:** Ongoing Randomized Clinical Trials in Cardiogenic Shock Management

Trial	Population	Study Design	Intervention	Comparator	Primary Outcome
PACCS (NCT05485376)	HF-related CS	Multicenter, randomized, adaptive	Early PAC-guided hemodynamic management	No or delayed PAC	In-hospital mortality
NORSHOCK (NCT05168462)	AMI-CS on norepinephrine	Multicenter, open-label RCT	Lower MAP target (>55 mmHg)	Standard MAP target (>65 mmHg)	Mortality, cost-effectiveness, RRT use
CAPITAL-DOREMI-2 (NCT05267886)	SCAI C–D CS	Multicenter, double-blind RCT	Inotrope therapy	Placebo	In-hospital mortality
LevoHeartShock (NCT04020263)	CS on vasopressors/inotropes	Double-blind RCT	Early levosimendan	Placebo	Composite morbidity–mortality
COCCA (NCT03773822)	Adult CS	Multicenter, double-blind RCT	Hydrocortisone + fludrocortisone	Placebo	Catecholamine-free days at 7 days
DOBERMANN (NCT05350592)	Pre-CS (SCAI B) post-AMI	Single-center, double-blind RCT	Low-dose dobutamine (24 h)	Placebo	Progression to CS
Istaroxime Pre-CS (NCT04325035)	Pre-CS, HFrEF	Multicenter, double-blind pilot RCT	Istaroxime infusion	Placebo	Hemodynamic improvement
CAPITOL MINOS (NCT05298124)	CS with ≥ moderate MR	Multicenter, open-label RCT	Transcatheter mitral repair	Medical therapy	Mortality, HF hospitalization
ULYSS (NCT05366452)	AMI-CS	Multicenter, open-label RCT	Early Impella CP before PCI	PCI + standard care	Clinical outcomes (mortality, MCS use)
UNLOAD-ECMO (NCT05577195)	Severe CS with severe LV dysfunction	Multicenter-randomized	Impella for LV unloading	VA-ECMO alone	Time to death from any cause within 30-days
ANCHOR (NCT04184635)	AMI-CS with congestion	Multicenter RCT	VA-ECMO + IABP LV unloading	Usual care	All-cause mortality

ANCHOR = Assessment of ECMO in Acute Myocardial Infarction Cardiogenic Shock; COCCA = Low Dose of Hydrocortisone and Fludrocortisone in Adult Cardiogenic Shock; DOBERMANN = Low-Dose Dobutamine and Single-Dose Tocilizumab in Acute Myocardial Infarction With High Risk of Cardiogenic Shock; DOREMI = Dobutamine Compared with Milrinone; HF = heart failure; HFrEF = heart failure with reduced ejection fraction; LevoHeartShock = Effect of Early Use of Levosimendan vs Placebo on Top of a Conventional Strategy of Inotrope Use on a Combined Morbidity-mortality Endpoint in Patients with CS; LV = left ventricle; MAP = mean arterial pressure; MR = mitral regurgitation; NORSHOCK = Clinical Outcome and Cost-effectiveness of Reduced Noradrenaline by Using a Lower Blood Pressure Target in Patients With AMI-CS; PACCS = PAC in CS; RCT = randomized controlled trial; RRT = renal replacement therapy; ULYSS = Evaluation of the Efficacy of Early Implantation of a Percutaneous LVAD in Acute Coronary Syndrome Complicated by CS Compared to Conventional Therapy; UNLOAD-ECMO = Left Ventricular Unloading to Improve Outcome in Cardiogenic Shock Patients on VA-ECMO; other abbreviations as in Tables 1, 3, 4, and 5.

Given no direct clinical trial evidence to support PAC use in CS, PACCS (PAC in CS; NCT05485376), a multicenter, randomized, adaptive trial is evaluating whether early invasive hemodynamic assessment with PAC in HF-CS reduces in-hospital mortality risk compared to no or delayed PAC use.^79^

Targeted hemodynamic parameters are critical in CS management. The commonly recommended mean arterial pressure ≥ 65 mmHg is extrapolated from sepsis trials or retrospective data.^80^ NORSHOCK (Clinical Outcome and Cost-effectiveness of Reduced Noradrenaline by Using a Lower Blood Pressure Target in Patients With AMI-CS; NCT05168462) is an open label, multicenter RCT comparing lower mean arterial pressure target (>55 mm Hg) vs standard >65 mm Hg in CS patients treated with noradrenaline, assessing mortality, cost-effectiveness, and use of renal replacement therapy.^81^ The DOREMI 2 trial (NCT05267886) is a multicenter, double-blind, placebo-controlled RCT enrolling SCAI stage C or D CS patients, comparing inotrope to placebo, with the primary outcome of in-hospital mortality and secondary safety outcomes.^82^

Levosimendan may offer better outcomes when combined with other therapies.^83^ LevoHeartShock (Effect of Early Use of Levosimendan vs Placebo on Top of a Conventional Strategy of Inotrope Use on a Combined Morbidity-mortality Endpoint in Patients with CS; NCT04020263) is a double-blind RCT evaluating early levosimendan use vs placebo in CS patients already on vasopressors and inotropes.^84^

Pre-CS (SCAI stage B), largely excluded from prior CS trials, represents a population in which impact of early intervention on progression to overt CS (SCAI stages C-E) is uncertain. Two trials are evaluating this question. The DOBERMANN (Low-Dose Dobutamine and Single-Dose Tocilizumab in Acute Myocardial Infarction With High Risk of Cardiogenic Shock; NCT05350592) is an investigator-initiated, double-blinded, placebo-controlled, randomized, single-center, clinical trial assessing 24 hours of dobutamine infusion vs placebo after PCI for AMI.^85^ Patients included are without initial CS and at an intermediate-high risk of in-hospital CS development based on risk scoring. Another inotropic medication, istaroxime, works by inhibiting the Na+/K + -ATPase activity (similar to cardiac glycosides) and activating the sarcoplasmic reticulum calciumATPase isoform 2a, thereby increasing inotropy.^86^ The Safety and Efficacy of Istaroxime for Pre-Cardiogenic Shock trial (NCT04325035) is a pilot, multicenter, randomized, double-blind, placebo-controlled study of istaroxime in patients with nonischemic pre-CS hospitalized for decompensated HF with persistent and clinically confirmed congestion and left ventricular ejection fraction ≤40%.^87^ The outcomes assessed are primarily hemodynamic, with laboratory and clinical secondary outcomes. Preliminary phase 2 study findings showed improved hemodynamic markers and general tolerance of the medication.^88^

There is a high prevalence of adrenal insufficiency in the broad critically-ill population; however, the utility of corticosteroid use in CS is not defined. COCCA (Low Dose of Hydrocortisone and Fludrocortisone in Adult Cardiogenic Shock; NCT03773822) is a multicenter, randomized, double blinded, placebo-controlled trial comparing intravenous hydrocortisone plus fludrocortisone with placebo in patients with CS.^89^ The primary outcome will be catecholamine-fee days after 7 days, with other secondary endpoints of all-cause mortality in predefined subgroups analyses are planned, including: postcardiotomy, myocardial infarction, etomidate use, and vasopressor use.

The CAPITOL MINOS (NCT05298124) is an open-label, multicenter, RCT assessing whether transcatheter mitral valve repair improves outcomes in CS patients with moderate or MR (SCAI stage C or D).^90^

Despite recent large clinical trials, many questions remain about tMCS use in CS. ULYSS (Evaluation of the Efficacy of Early Implantation of a Percutaneous LVAD in Acute Coronary Syndrome Complicated by CS Compared to Conventional Therapy; NCT05366452) is an ongoing randomized, multicenter, and prospective open-label study evaluating the efficacy of the early implantation of IMPELLA CP support before culprit lesion PCI compared to standard PCI and usual care.^91^ The ANCHOR (Assessment of ECMO in Acute Myocardial Infarction Cardiogenic Shock; NCT04184635) trial will assess difference in mortality in patients with AMI-CS and signs of congestion by randomizing to VA-ECMO plus left ventricular unloading with IABP vs usual care.^92^ Lastly, the ongoing UNLOAD-ECMO (Left Ventricular Unloading to Improve Outcome in Cardiogenic Shock Patients on VA-ECMO; NCT05577195) trial is evaluating whether early active unloading with Impella added to VA-ECMO improves 30-day survival compared with VA-ECMO alone in patients with severe CS.^93^

**Figure 1:**
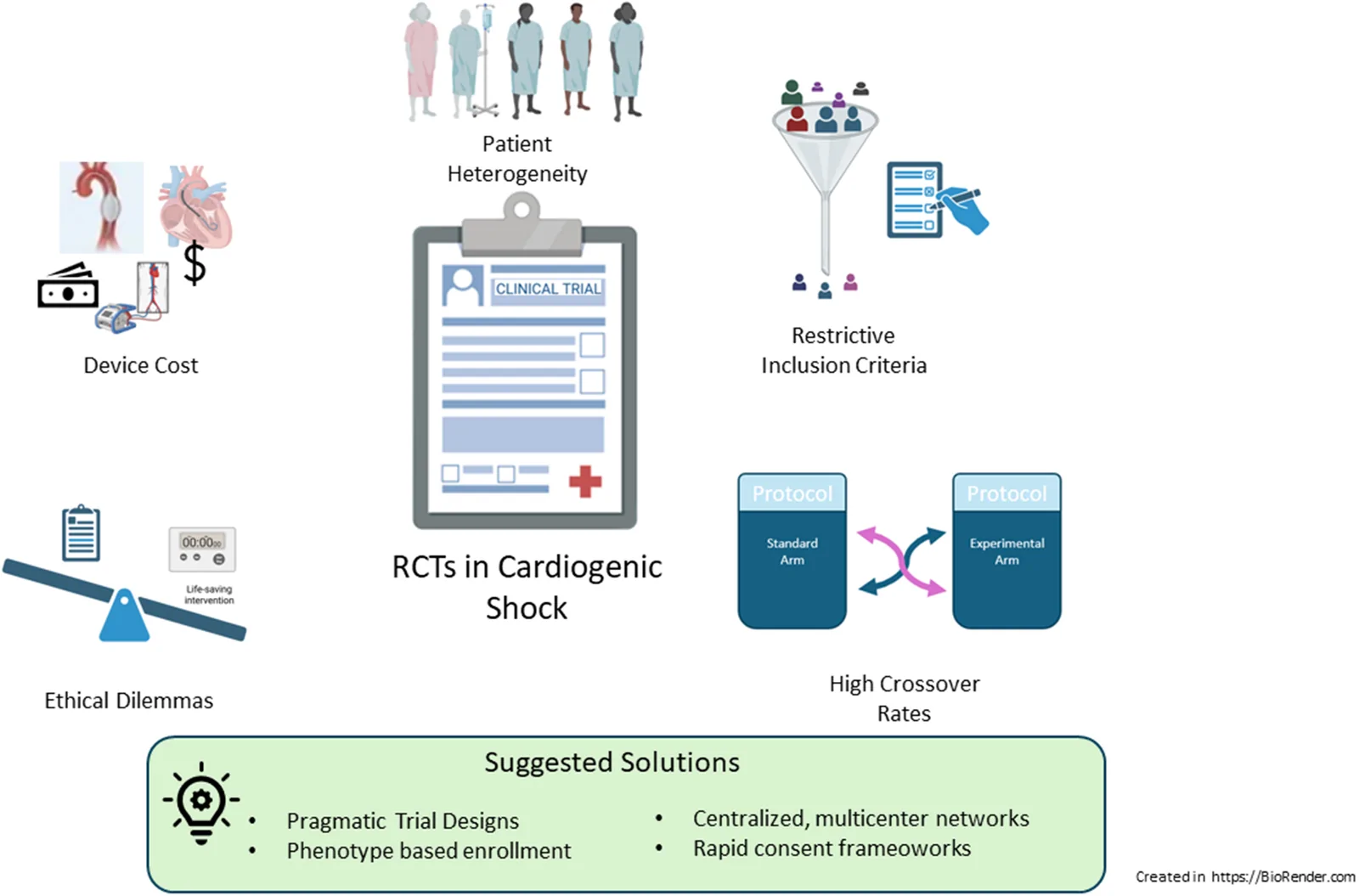
The Enrollment Challenges in Clinical Trials in Cardiogenic Shock RCT = randomized controlled trial.

However, RCTs in CS face significant enrollment challenges (Figure 1), which can impact their timely completion.^94^ These challenges are inherent to a heterogeneous, critically ill population where restrictive inclusion criteria introduce selection bias and illness severity drives high crossover rates. Ethical considerations surrounding consent, coupled with risk of delaying lifesaving interventions, further complicate trail conduct. In addition, device-based trials introduce cost and funding constraints, often necessitating industry involvement. Addressing these barriers will require pragmatic trial designs, streamlined enrollment strategies, and phenotypic-specific approaches.

## Conclusions

Despite decades of clinical experience, CS remains a highly heterogenous syndrome with persistently high mortality and a limited base of definitive randomized evidence. Emerging data from contemporary trials, registries, and observational studies suggest that physiology driven strategies integrating PAC use, targeted pharmacological therapy, selective use of tMCS, and structural interventions may improve outcomes in carefully selected patient. Although current guidelines provide a foundational framework of CS management, RCTs also highlight substantial uncertainty regarding patient selection, timing and escalation of therapy, which underscores a growing gap between evolving clinical practice and existing guideline recommendations. Advancing CS care will require updated, phenotype-based guidelines informed by pragmatic, adequately powered RCTs that reflect real world complexity, to meaningfully improve outcomes.

Perspective **COMPETENCY IN PATIENT CARE AND PROCEDURAL SKILLS:** Contemporary CS management must transition from fixed algorithm-driven approaches toward individualized, phenotype-driven care. Effective CS management requires seamless integration of comprehensive invasive hemodynamic assessment to precisely differentiate underlying shock phenotypes (isolated left-ventricular, right-ventricular, or biventricular failure) and identify relevant hemodynamic targets. These parameters help inform the selection and timing of vasoactive medications, and tMCS devices, while balancing anticipated hemodynamic benefits against treatment related complications. Standardized, protocol-driven care pathways delivered via multidisciplinary shock teams may facilitate timely escalation of pharmacologic and tMCS before the onset of irreversible, multiorgan failure.

**TRANSLATIONAL OUTLOOK:** Future CS research needs to move away from head-to-head comparisons of individual drugs or isolated devices towards evaluation of comprehensive treatment strategies. Advancing the field requires pragmatic, and adaptive platform trials embedded within multidisciplinary shock networks. These trials should integrate early phenotypic stratification, predefined hemodynamic goals, precise timing of intervention, and structured escalation pathways to identify which therapies benefit specific patient phenotypes at different shock stages.

## Funding support and author disclosures

Dr Narang has received speaker fees from Boehringer Ingelheim and AstraZeneca; and has received consulting fees from Alnylam and BridgeBio. Dr Bhardwaj serves on CEA committee for Baim Clinical Research Institute; and has served on advisory board for Johnson & Johnson. All other authors have reported that they have no relationships relevant to the contents of this paper to disclose.
